# Bioinformatics Analysis of the Tomato *SlPR5* Gene Family and the Thaumatin-like Protein SlPR5-3 Positively Regulates Tomato Resistance to *Pst* DC3000

**DOI:** 10.3390/plants14213389

**Published:** 2025-11-05

**Authors:** Xinyue Pang, Yue Wang, Binyu Jiang, Dalong Li, He Zhang, Dong Liu, Xiangyang Xu, Tingting Zhao

**Affiliations:** 1 Tomato Research Institute, College of Horticulture and Landscape Architecture, Northeast Agricultural University, Harbin 150030, China; pxinyue1201@163.com (X.P.); yuewang20241015@163.com (Y.W.); jiangbinyu2001@163.com (B.J.); lidalong@neau.edu.cn (D.L.); esculentum@neau.edu.cn (H.Z.); 2 Key Laboratory of Biology and Genetic Improvement of Horticultural Crops (Northeast Region), Ministry of Agriculture and Rural Affairs, Northeast Agricultural University, Harbin 150030, China; 3 College of Plant Protection, Northeast Agricultural University, Harbin 150030, China; dongliu@neau.edu.cn

**Keywords:** tomato, *SlPR5* family, *Pst* DC3000

## Abstract

Studies on how *SlPR5* genes are involved in *Pst* DC3000 disease resistance response are lacking. Here, 27 members of the tomato *SlPR5* gene family were identified and analyzed. Analysis of conserved structural domains and promoter structure revealed that *SlPR5* family members are structurally conserved and contain a variety of antidisease and antistress response elements. We screened out *SlPR5-3*, which was significantly upregulated, through an analysis of the expression pattern of *Pst* DC3000 in tomato after inoculation. We generated *SlPR5-3* mutants via CRISPR/Cas9 gene editing and *SlPR5-3-*overexpressing tomato plants to elucidate the function of this gene. The results showed that the *SlPR5-3* overexpression lines had reduced lesions, significantly lower pathogen counts, and significantly higher activity indexes of defense-related enzymes, while the mutant lines showed the opposite, indicating that the *SlPR5-3* gene positively regulates the immune response against *Pst* DC3000 in tomato. In this study, we systematically mined and analyzed the tomato *SlPR5* family genes, screened out the important genes in this family for the regulation of *Pst* DC3000 disease resistance, and verified the disease resistance regulatory function of *SlPR5-3*, laying the foundation for the theoretical study of tomato *Pst* DC3000 disease resistance and providing a new molecular target for the future breeding of tomato disease resistance.

## 1. Introduction

As a valuable horticultural cash crop and an important part of the human diet, tomato (*Solanum lycopersicum*) is one of the main vegetable crops in China. A variety of factors affect the yield and quality of tomato [1], and disease is among the major constraints to tomato production. Bacterial leaf spot of tomato, also known as spot or blotch disease, is a very serious bacterial disease caused by *Pseudomonas syringae pv. tomato* DC3000 (*Pst* DC3000) [2]. Bacterial leaf spot may occur throughout the reproductive period of tomato, and it mainly affects the leaves, stems, flowers, fruit stalks, and fruits [3]. In addition to fungal and viral diseases, bacterial diseases have a major impact on plants. The prevalence of bacterial diseases of vegetables is increasing annually, and these diseases occur more frequently in cruciferous, cucurbit, lycopene and leguminous crops, which are common economic crops, resulting in substantial economic losses. Bacterial diseases are widespread and can easily spread over a large area when conditions are favorable, resulting in massive yield loss and field destruction; thus, bacterial control needs to be emphasized during production. Currently, disease control relies mainly on chemical pesticides, but the frequent use of pesticides poses a serious threat to food safety and the ecological environment.

Typical tomato disease resistance responses to *Pst* DC3000 include PAMP-triggered immunity (PTI) and effector-triggered immunity (ETI). When *Pst* DC3000 infects tomato leaves [4], the 22-amino-acid peptide of the N-terminal end of the bacterial flagellin flg22 and its derivatives flgII-28 activate the pattern recognition receptors (PRRs) Fls2 and Fls3, respectively [5,6,7], triggering early PTI responses such as reactive oxygen species (ROS) production, activation of the mitogen-activated protein kinase (MAPK) cascade, and transcriptional reprogramming of a subset of defense genes [8,9,10]. Two *Pst* effector proteins, AvrPto and AvrPtoB [11,12], subsequently bind and interfere with the binding of the intracellular protein kinase structural domains of Fls2, Fls3, and the coreceptor Bak1, thereby disrupting the host response to these microbe-associated molecular patterns (MAMPs) [13]. Moreover, these two effector proteins are recognized by the host Pto kinase and activate ETI via the NLR protein Prf [14,15].

The regulatory network involved in plant disease resistance to *Pst* DC3000 is very complex, and recent studies have shown that a variety of biological processes and different types of regulatory factors are involved in this network. Transcription factors, such as the Pto interaction protein, Pti4 and Pti5 [16], which belong to the ERF (ethylene-response factor) family, play important roles and can improve resistance to bacterial leaf spot in *Arabidopsis* and tomato [17]. *AtWRKY1* can bind to the promoter of the disease resistance-associated protein AtPR1 to repress its transcription and thus negatively regulate *Arabidopsis* resistance to *Pst* DC3000 [18]. The expression of the tomato bHLH transcription factor SlNrd1 protein increases tomato susceptibility to *Pst* DC3000 by repressing the expression of the defense gene *SlAgp1* [19]. Mutation of the *Arabidopsis* transcription factor AtREPLUMLESS (AtRPL) decreases the expression of the *AtGH3* promoter, resulting in the accumulation of indole-3-acetic acid aspartate (IAA-Asp) and the activation of the transcription of virulence effector genes. In addition to transcription factors, many other types of proteins and regulatory pathways are involved in the regulation of *Pst* DC3000 resistance; e.g., the tomato sugar transporter protein SlSTP2 is induced by *Pst* DC3000 and positively regulates tomato resistance to bacterial leaf spot, and SlSTP2 interacts with the positive regulator SlAGB1 [20]. The histone deacetylase HDA6 reduces *Arabidopsis* resistance to *Pst* DC3000 by inhibiting the biosynthesis of salicylic acid [21]. As research continues to expand and deepen, more details of the regulation of the plant response to *Pst* DC3000 disease resistance are being revealed, but the regulatory processes still need to be explored in depth to elucidate the basic regulatory network.

Disease course-associated proteins compose a general class of proteins whose production is induced by biotic or abiotic stresses in plants [22]. A protein associated with allergic reactions was detected for the first time in tobacco leaves infected with tobacco mosaic virus (TMV) and referred to as a disease process-related protein, abbreviated as PR or PRP [23]. With the in-depth study and development of disease course-related proteins, PR proteins were categorized into 17 families, distributed as PR-1~17 [24]. Studies on the role of PR proteins in plant fruiting and stress tolerance have focused on PR-1, PR-2, PR-5, and PR-10. These classes of proteins have antimicrobial activity, with PR-5 exhibiting antifungal activity and resistance to abiotic stresses [25]. SlPR5 family proteins are called thaumatin-like proteins (TLPs) because of their high amino acid sequence homology to thaumatin of the African species *Thaumatococcus daniellii L*. The TLPs are also known as “sweet taste proteins” [26]. SlPR5 proteins are unique proteins with multiple functions, such as antimicrobial activity; they are also associated with plant stress tolerance, such as increased tolerance to frost [27] and enhanced tolerance to salt stress and drought stress [25]. The SlPR5 family is an important family of disease process-associated proteins in the plant kingdom that are associated with responses to biotic stresses. Twenty-seven *SlPR5* family members have been identified in the tomato genome, and PR5 genes have been characterized in many species other than tomato; 43 PR5 members have been identified in the *Setaria italica* genome [28], 32 in *Allium sativum* [29], 42 in *Glycine max* [30], and 31 in *Camellia sinensis* [31], and some PR5 members exhibit broad-spectrum resistance to a variety of pathogens. For example, the *PR5-x* gene plays a role in tomato resistance to *Fusarium oxysporum,* and the accumulation of the PR-P2 protein is associated with disease resistance [32]. NP24 I and NP24 II were found to inhibit the growth of *Fusarium* beetles and *Trichoderma ferruginea* in ripe tomato fruits [33]. Similarly, exudin-like PR-5 proteins not only are tolerant to abiotic stresses in plants but also have a wide range of antifungal activities [34]. In addition, thaumatin-like protein increased the resistance of tomato to five fungal pathogens and bacterial pathogens by increasing the activity of β-1,3-glucanase [35]. *Pst* DC3000 infestation of tomato induced a significant up-regulation of *SlPR5* gene expression through activation of the salicylic acid-mediated pathway [36,37,38]. Up-regulation of PR5 expression in Arabidopsis improves resistance to Bacterial Leaf Spot [39]. Although the roles of *SlPR5* genes in disease resistance in tomato have been described, little has been reported on their roles in bacterial leaf spot resistance in tomato.

In view of the important role of the *SlPR5* family of genes in plant disease resistance, this study utilized bioinformatics techniques for the genome-wide identification and functional prediction of the *SlPR5* gene family in tomato. A preliminary analysis of the motif composition and promoter cis-elements was carried out to clarify the structural basis of the disease resistance of this family of genes. Further functional prediction was carried out on the basis of the RNA-seq data via expression pattern analysis, after which the *SlPR5* genes were determined to have significant differential expression patterns under biotic stress (*Pst* DC3000) and in specific tissues. Finally, the *SlPR5-3* gene with the most important role was predicted at the genome level and transcript level, and its function was verified by experimental analysis. This study provides a molecular target (*SlPR5-3*) for the genetic improvement of tomato resistance to bacterial leaf spot.

## 2. Materials and Methods

### 2.1. Identification and Evolutionary Tree Construction of SlPR5 Gene Family Members in Tomato

Tomato genome and gene annotation files were downloaded from the Solanaceae Genomics Network (https://solgenomics.net/, accessed on 3 September 2023). website [40]. A hidden Markov model (HMM E-value < 1 × 10^−5^) (http://hmmer.janelia.org/, accessed on 5 September 2023) of the conserved structural domain of *SlPR5* (No. PF 00314) was constructed from the Pfam database (http://pfam.xfam.org/family, accessed on 11 September 2023) for the identification of all possible *SlPR5* members of the tomato genome in the transcription factor family [41]. Further screening of tomato SlPR5 family members was carried out using NCBI-BLASTP (https://blast.ncbi.nlm.nih.gov/Blast.cgi, accessed on 16 September 2023) (BLAST E-value < 1 × 10^−5^~1 × 10^−10^). Finally, sequence analysis of tomato SlPR5 family members was carried out using NCBI-CDD (https://www.ncbi.nlm.nih.gov/cdd/, accessed on 20 September 2023) to confirm the members of the tomato *SlPR5* gene family. Nucleotide sequences (https://sgn.cornell.edu/help/index.pl/, accessed on 23 September 2023) and amino acid sequences of 27 tomato *SlPR5* candidate genes were downloaded from the Ensembl Plants database (http://plants.ensembl.org/index.html/, accessed on 24 September 2023) for subsequent analysis [42]. The 27 conserved structural domains of the tomato SlPR5 protein were validated by the SMART database (http://smart.embl-heidelberg.de/, accessed on 27 September 2023). The *Arabidopsis* SlPR5 protein sequence was obtained from The Arabidopsis Information Resource (TAIR) (http://www.arabidopsis.org/, accessed on 28 September 2023) [43]. SlPR5 protein sequences from tomato and *Arabidopsis* were subjected to multiple sequence comparisons by ClusterX. On the basis of the comparison results, a rootless developmental tree was constructed using MEGA 7 with the neighbor-joining (NJ) method.

### 2.2. Analysis of the Physicochemical Properties of the SlPR5 Family Members in Tomato

The amino acid number, relative molecular weight and theoretical isoelectric point of SlPR5 family members were predicted by ExPASy’s ProtParam tool (https://web.expasy.org/protparam/, accessed on 30 September 2023) [44]. Signal peptides of 27 tomato SlPR5 proteins were predicted using SignalP 4.1 (http://www.cbs.dtu.dk/services/SignalP/, accessed on 8 October 2023) [45] and WoLF PSORT (https://wolfpsort.hgc.jp/, accessed on 11 October 2023) to predict their subcellular localization [46].

### 2.3. Chromosomal Localization Prediction, Gene Structure and Conserved Structural Domain Analysis of SlPR5 in Tomato

The annotation information of 27 tomato *SlPR5* genomes was retrieved from SGN (https://sgn.cornell.edu/help/index.pl, accessed on 13 October 2023), and the chromosomal position of tomato *SlPR5* was obtained from MG2C (http://mg2c.iask.in/mg2c_v2.0/, accessed on 15 October 2023). The structure and conserved structural domains of the tomato *SlPR5* genes were analyzed via NCBI-CDD and visualization in TBtools [47]. To further clarify the functions of *SlPR5* genes, the protein sequence was submitted to MEME (http://meme-suite.org/tools/meme, accessed on 18 October 2023) [48] to characterize the order of the *SlPR5* genes in tomato. The maximum number of motifs was set to 10, and the other parameters were set to default values.

### 2.4. Analysis of Cis-Regulatory Elements

The 2000 bp upstream promoter sequences of 27 candidate genes were downloaded from the Tomato Genome Database (https://solgenomics.net/, accessed on 19 October 2023) and submitted to Plant-CARE (https://bioinformatics.psb.ugent.be/webtools/plantcare/html/, accessed on 21 October 2023) for the prediction of cis-acting elements in the promoter regions.

### 2.5. Subcellular Localization of the SlPR5-3 Protein

Primer Premier 5.0 software was used to design primers for *SlPR5-3*-EGFP (Table S1) to amplify *SlPR5-3* with the stop codon removed. The pCAMBIA2300-EGFP vector was enzymatically digested with *BamHI* and *EcoRI*, the target fragment was recombined with the plasmid, the recombinant plasmid was transfected into *Escherichia coli*, and the plasmid was transfected into *Agrobacterium* for the infection of tobacco plants. After 3 days of incubation in the dark, fluorescence imaging was performed by laser coaggregation microscopy.

### 2.6. Plant Material and Treatment

*Ailsa Craig* (AC) tomato material was used as the test material, which is susceptible to disease. All seedlings were grown in a thermostat incubator at a diurnal temperature of 20–25 °C with 16 h of light and 8 h of darkness, a light intensity of 35,000 lx, and a relative humidity of 45%. When the tomato seedlings reached the five-leaf stage, they were subjected to foliar sprays of *Pst* DC3000 and AC plant stress treatments, followed by qRT–PCR. Specifically, *Pst* DC3000 was cultured on King’s B (KB) medium supplemented with 50 mg/L rifampicin overnight until the OD600 was 0.6 to 0.8 (OD 0.1 = 10^8^ cfu mL^−1^). Then, *Pst* DC3000 was diluted with 10 mM MgCl_2_ to a concentration of 10^7^ cfu mL^−1^. Before infiltration, *Pst* DC3000 was resuspended in 10 mM MgCl_2_ to an OD600 of 0.0002 [4]. Ten seedlings were selected for each treatment, and all the treatments were repeated three times. Leaves from *SlPR5-3* overexpression and CRISPR mutant plants were collected at 0, 12, 24, 48, and 72 h after pathogen stress for subsequent determination of physiological indices, immediately frozen in liquid nitrogen and stored at −80 °C.

### 2.7. Transcriptome Data Analysis

The expression of *SlPR5* family members after inoculation with *Pst* DC3000 was queried through the Tomato Gene Function Database (http://ted.bti.cornell.edu/, accessed on 22 December 2023). A tissue-specific analysis of *SlPR5* family members in tomato was performed through Plant Biology’s Bioanalytical Resources (http://bar.utoronto.ca/eplant/, accessed on 24 December 2023). An expression heatmap was constructed using the TBtools heatmap.

### 2.8. Real-Time Quantitative PCR (qRT–PCR) Analysis

Total RNA was extracted from the samples using TRIzol. cDNA synthesis was performed using an M-MLVRTase kit (TaKaRa, Dalian, China). qRT–PCR was performed using the iQ 5 system. The actin-7 gene (Solyc11g005330.1.1) was used as an internal control (Table S2). For each qRT–PCR, the mixture consisted of 10 μL of SYBR^®^ Green Master Mix, 0.5 μL of forward and reverse primers, 1 μL of diluted cDNA, and 8 μL of ddH_2_O. In a 20 μL reaction, the reaction was carried out as follows: 95 °C for 5 min, followed by 40 cycles of 94 °C for 5 s, 60 °C for 15 s, and 72 °C for 10 s; three biological replicates were included.

### 2.9. Measurement of Physiological Indicators

Leaf samples of WT, OE-*SlPR5-3*, and Cr-*SlPR5-3* plants were collected before and after pathogen infection, and each sample consisted of three replicates. Superoxide dismutase (SOD) and peroxidase (POD) activities as well as catalase (CAT) activity and malondialdehyde (MDA) content were determined according to the instructions provided with the kit used (Suzhou Grace Biotechnolgy Co., Ltd, Suzhou, China).

### 2.10. Construction and Validation of SlPR5-3-Overexpressing and CRISPR Mutant Lines

Primer Premier 5.0 software was used to design primers for the *SlPR5-3* gene, clone the CDS of the *SlPR5-3* gene, insert the CDS into the pCAMBIA2300-HA vector, and construct the overexpression plasmid. Overexpressing plants were obtained by *Agrobacterium*-mediated transformation, and then the overexpressing lines were screened with kanamycin and verified by genomic PCR and qRT–PCR. CRISPR mutant vectors were constructed, and targets were designed using the TargetDesign program on the CRISPR-GE web page (http://skl.scau.edu.cn/, accessed on 3 January 2024). The sgRNA expression cassettes for the two targets of each gene were constructed by using pYLgRNA-AtU3b and pYLgRNA-AtU3d as templates, amplified by the overlap method, and then ligated into the pYLCRISPR/Cas9P35S-H binary vector, the details of which are referenced from the operation published by Zeng, Dong-chang et al. [49].

### 2.11. Reactive Oxygen Species Staining

Reactive oxygen species (ROS) were detected using nitrogen blue tetrazolium (NBT) [50] and 3,3′-diaminobenzidine (DAB) staining [51]. Leaves of wild-type plants, *SlPR5-3*-overexpressing plants, and *SlPR5-3* CRISPR mutant plants 3 days after inoculation with *Pst* DC3000 were immersed in 3,3′-diaminobenzidine (DAB) and nitroblue tetrazolium (NBT) dye solutions. After incubation in the dark for 12 h, the dye solutions were discarded, 30 mL of anhydrous ethanol was added, and the material was heated in a water bath at 100 °C for 12 min while shaking every 3 min. If the color of the leaves did not fade completely, they were washed again several times with anhydrous ethanol. The fully discolored leaves were placed on slides to observe the staining and collect images.

### 2.12. Statistical Analysis

SPSS 24.0 (IBM) and prism 8.0 (GraphPad) software were used for statistical analysis. Student’s t-test (*p* < 0.05) and least significant difference (LSD) tests (*p* < 0.05) were performed to analyze the significant difference. All measurements were taken from the average of at least three independent biological replicates.

## 3. Results

### 3.1. Identification and Physical Characterization of the SlPR5 Family in Tomato

On the basis of HMM and conserved structural domain analysis, a total of 27 SlPR5 family members were identified in tomato, and their basic information is listed in Table 1. The predicted relative molecular weights of the proteins ranged from 8266.26 Da (SlPR5-21) to 97,494.25 Da (SlPR5-8); SlPR5-21 (75 aa) had the shortest protein sequence, and SlPR5-8 (878 aa) had the longest protein sequence. By predicting the physicochemical properties, we found that eleven of them were basic proteins (PI > 7) and the remaining 16 were acidic proteins (PI < 7). In addition, nine proteins of the SlPR5 family have a GRAVY index higher than 0, and the other SlPR5 proteins were hydrophilic. Among them, the minimum GRAVY index was 46.29 (SlPR5-19), and the maximum was 79.96 (SlPR5-15). Seventeen of the 27 SlPR5 family members were stable proteins (instability coefficient < 40), and the rest were unstable. As shown in Table 1, most members of the SlPR5 family are located in the cell wall and chloroplasts, with a few in other locations. The exact locations of these proteins require further experimental verification. The locus IDs for the tomato SlPR5 family are in Table S4. 

### 3.2. Analysis of Protein Conserved Motifs and Structural Domains

**Figure 1:**
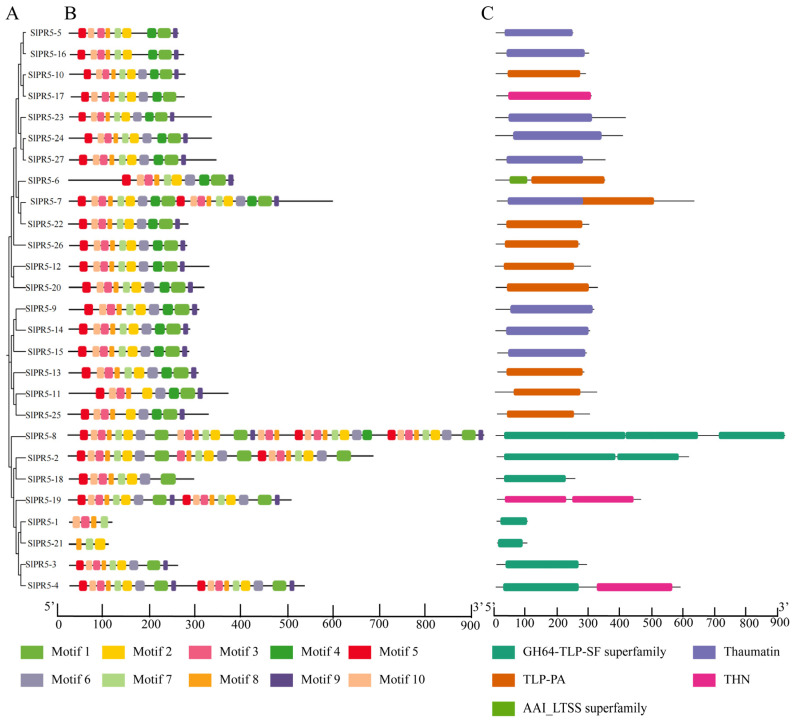
NJ tree, motif and domain of SlPR5 in tomato. (**A**), NJ tree constructed by MEGA 7.0 software using SlPR5 family members. (**B**), Motif positions of tomato SlPR5 family members. The ten motifs are displayed in different colors. (**C**), Domain positions of tomato SlPR5 family members. The scale at the bottom shows the length of the motif and domain.

A phylogenetic tree containing only the SlPR5 family was constructed using MEGA-X, which classified the SlPR5 family into three groups (Figure 1A), and conserved motifs and structural domains were analyzed for SlPR5 family members (Figure 1B,C). The results of the motif analysis revealed a total of 10 conserved motifs in the tomato SlPR5 family proteins. Among them, motif 2 and motif 8 were conserved in every SlPR5 family protein (Figure 1B). Analysis of the conserved structural domains of SlPR5 family proteins revealed that nine SlPR5 contained a conserved thaumatin structural domain, 10 SlPR5 contained a conserved TLP-PA structural domain, and seven SlPR5 contained a conserved GH64-TLP-SF structural domain (Figure 1C). All of these structural domains are associated with pathogen defense, and the SlPR5 family may play a regulatory role in the disease resistance pathway in tomato.

### 3.3. Chromosome Localization Prediction and Cis-Acting Element Analysis

**Figure 2:**
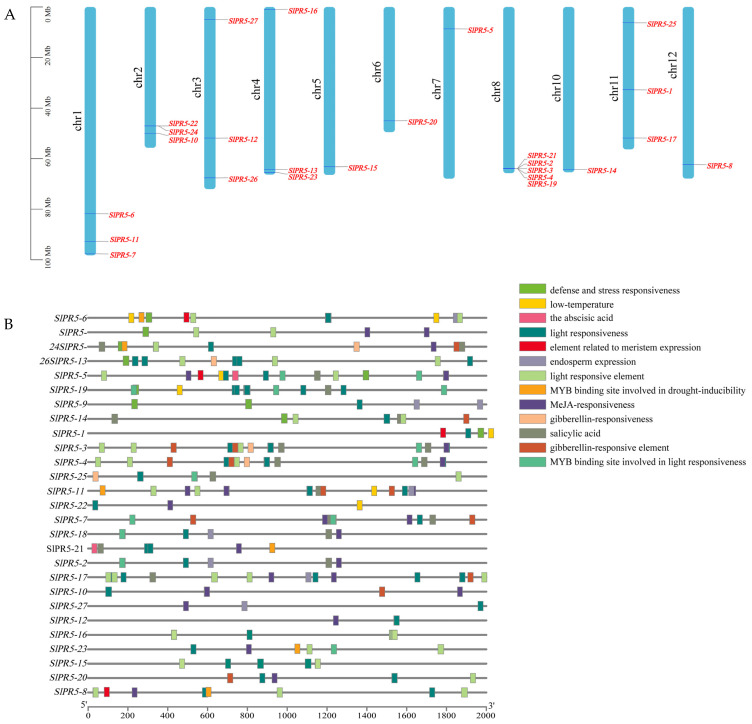
Chromosomal mapping and cis-acting elements of the *SlPR5* genes in tomato. (**A**) The chromosome number is located to the left of each chromosome, and the scale value on the left corresponds to the chromosome length. (**B**) The different colored squares represent different cis-acting elements.

Using tomato genome annotation information and TBtools software (v2.034), we visualized the chromosomal distribution of family members of the tomato *SlPR5* gene family (Figure 2A). We found that tomato *SlPR5* genes were unevenly distributed on the chromosomes, with chromosome 8 containing the greatest number of genes, five in total, and showing coaggregation, which may be related to tandem replication of the chromosomes.

To investigate the function of the tomato *SlPR5* gene, the promoter sequence (2000 bp upstream of the CDS) of the tomato *SlPR5* gene was analyzed to detect cis-elements (Figure 2B) using Plant-CARE. A total of 13 major classes of cis-acting elements were detected in the promoter regions of members of the tomato *SlPR5* gene family. These include abiotic stress response elements in response to light, salt stress, and drought and low-temperature stress and hormone (ABA, GA, MeJA, and SA) response elements associated with disease resistance. These results suggest that tomato *SlPR5* genes may play roles in tomato growth and development as well as in biotic and abiotic stress responses.

### 3.4. Analysis of SlPR5 Family Gene Expression Patterns in Tomato on the Basis of Transcriptome Data

**Figure 3:**
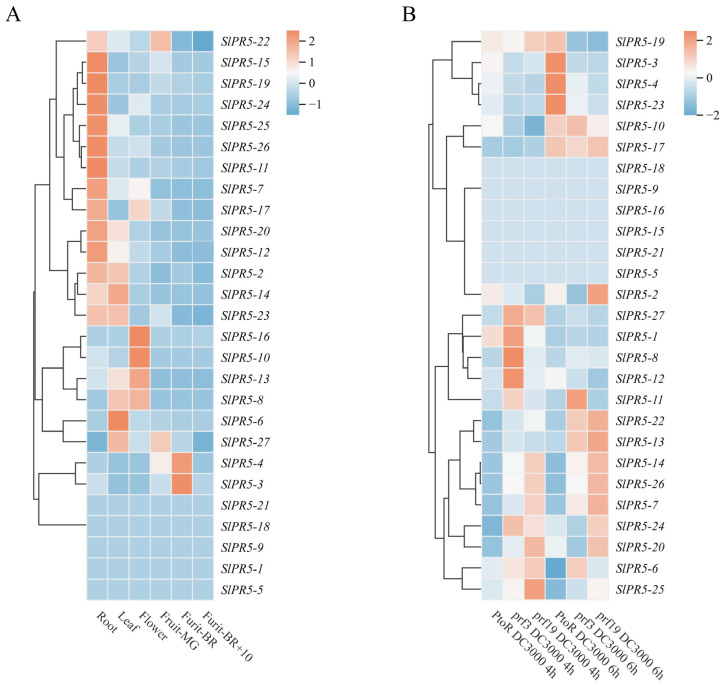
Expression of *SlPR5* genes. (**A**) The chromaticity on the right side of the heatmap shows the relative expression, and the color gradient from blue to red corresponds to an increase in expression. Fruit_MG indicates mature green (MG), Fruit_BR indicates a breaker (early ripening), and Fruit_BR +10 indicates 10 days post-B (red ripe). (**B**) Number of *SlPR5* family members after *Pst* DC3000 inoculation. The chromaticity on the right side of the heatmap represents the relative expression, and the color gradient from green to red corresponds to an increase in expression. Note: PtoR is a disease resistant variety, prf is a susceptible variety.

To investigate the role of the *SlPR5* gene family in various tomato tissues at different growth stages, we analyzed the expression levels of these genes in six tomato tissues, through Plant Biology’s Bioanalytical Resources (http://bar.utoronto.ca/eplant/, accessed on 15 October 2023) (Figure 3A). Among them, 14 genes had the highest expression in roots; 9 genes had the highest expression in leaves. Compared with the other genes, *SlPR5-3* and *SlPR5-4* had the highest expression in tomato fruits at the turn-color stage (Figure 3A). It is hypothesized that this gene may be closely related to plant resistance and growth and development. The expression of *SlPR5* family members after inoculation with *Pst* DC3000 was queried through the Tomato Gene Function Database, and the expression of *SlPR5-3*, *SlPR5-4*, *SlPR5-10*, *SlPR5-17*, *SlPR5-19*, and *SlPR5-23* significantly increased in disease-resistant tomato materials 6 h after inoculation (Figure 3B); these genes might be involved in the regulation of disease resistance in tomato.

### 3.5. Analysis of the Disease Resistance Response of SlPR5 Genes to Pst DC3000 and the Tissue Specificity of SlPR5-3

**Figure 4:**
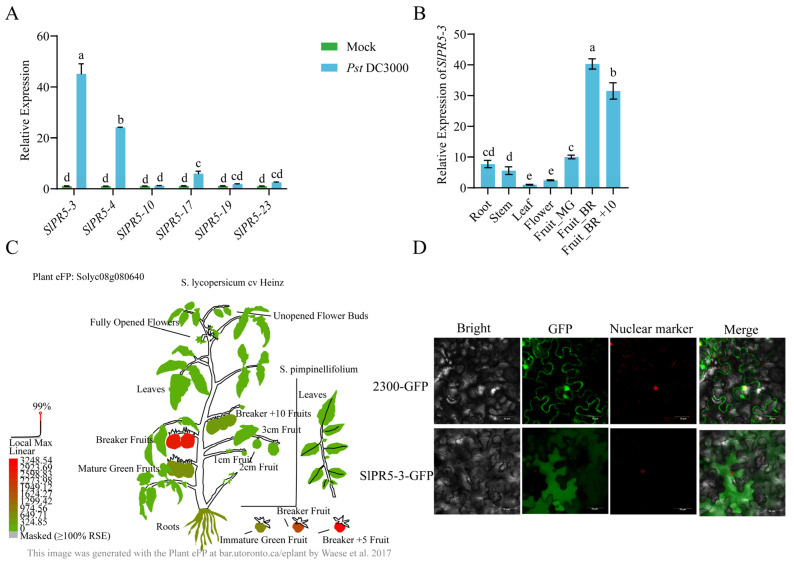
Analysis of tissue expression patterns of *SlPR5-3*. (**A**) Number of *SlPR5* family members in ‘Ailsa Craig’ tomato after *Pst* DC3000 inoculation. (**B**) Tissue-specific expression. Fruit_MG indicates mature green (MG), Fruit_BR indicates breaker fruits (early ripening), and Fruit_BR +10 indicates 10 days post-B (red ripe). Letters (a–e) indicate significant differences (*p* ≤ 0.05). (**C**) Graphical representation of the *SlPR5-3* gene content in various tissues of tomato plants [52]. (**D**) Results of the subcellular localization of *SlPR5-3*.

Based on the transcriptome data, it was observed that the expression of six genes was significantly up-regulated in the disease-resistant varieties. To explore the response patterns of the above six *SlPR5* genes in susceptible tomato varieties inoculated with *Pst* DC3000, we used ‘*Ailsa Craig* (AC)’ tomato as a material and determined the expression of *SlPR5* members in plants inoculated with *Pst* DC3000 at 0 h and 24 h (Figure 4A). The expression levels of the *SlPR5-10, SlPR5-17*, and *SlPR5-22* genes were not significantly upregulated at 24 h compared with those at 0 h, whereas the expression levels of *SlPR5-3, SlPR5-4*, and *SlPR5-23* were significantly upregulated, with the greatest upregulation occurring in *SlPR5-3*. These results suggest that *SlPR5-3* gene expression is induced by *Pst* DC3000 and that this gene may play an important role in tomato resistance to bacterial leaf spot. Therefore, We chose the *SlPR5-3* gene with the highest expression for functional validation, and the others may continue to be analyzed in future studies. A phylogenetic comparison of SlPR5-3 with other Solanaceae species was also made (Figure S1).

To investigate the transcript levels of *SlPR5-3* in different tissues, the expression of *SlPR5-3* in different tissues of tomato was predicted using an expression prediction website and verified by qRT–PCR (Figure 4B,C). The results revealed that the *SlPR5-3* gene was expressed in all the tissues, with higher and gradually increasing expression in Fruit_MG, Fruit_B, and Fruit_B +10, with the highest expression in Fruit-B and lower expression in the leaves and flowers (Figure 4B,C). The above results indicated that *SlPR5-3* gene expression differed significantly among the different tissues and was most highly expressed in the BR_+10 fruits. Thus, the *SlPR5-3* gene may play an important role in tomato fruit ripening.

To clarify the site of action of *SlPR5-3* in plant cells, the subcellular localization of *SlPR5-3* was determined. As shown in Figure 4D, the *SlPR5-3*-GFP fusion protein was distributed in the cytoplasm when expressed, indicating that the SlPR5-3 protein is localized in the cytoplasm.

### 3.6. Generation of SlPR5-3 Mutants and Overexpression Plants

**Figure 5:**
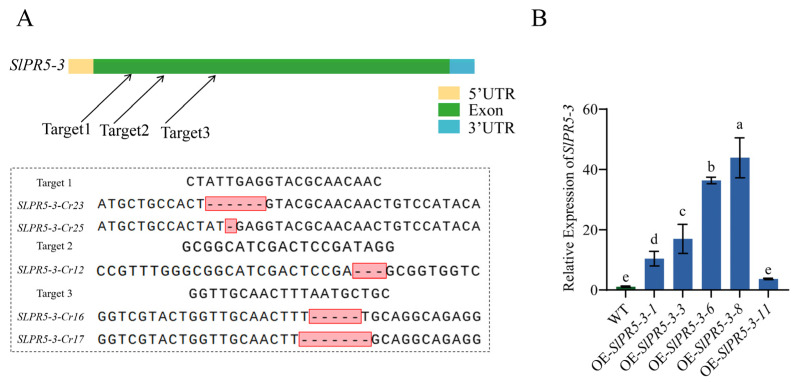
*SlPR5-3* identification in positive plants of the overexpression and CRISPR mutation lines. (**A**) Sequencing results of the T1 generation of *SlPR5-3* gene CRISPR mutant lines; (**B**) Identification of *SlPR5-3*-overexpressing lines. Letters (a–e) indicate significant differences (*p* ≤ 0.05).

In the identification of *SlPR5-3* mutants and overexpression plants, the genome is amplified with primers flanking the target site, and the amplified PCR product is sequenced. From the sequencing results, the Cr-12, Cr-16, Cr-17, Cr-23, and Cr-25 lines produced 3 bp, 5 bp, 7 bp, 6 bp, and 1 bp fragment deletions, respectively (Figure 5A), and three lines, Cr-16, Cr-17, and Cr-23, were selected for the subsequent experiments. The expression of *SlPR5-3* in these five lines was examined by qRT–PCR of the positive plants, and the results revealed that the expression of the *SlPR5-3* gene was approximately 10-fold, 17-fold, 36-fold, and 44-fold greater in the four *SlPR5-3*-overexpressing lines than in the wild-type line (Figure 5B). All the above four lines were available for subsequent experiments, and full progeny seeds from the four lines OE-3, OE-6 and OE-8 were selected for subsequent studies. All the overexpression plants tested were T2 generation plants.

### 3.7. Phenotypic Analysis of Disease Resistance in SlPR5-3-Overexpressing and Mutant Lines

**Figure 6:**
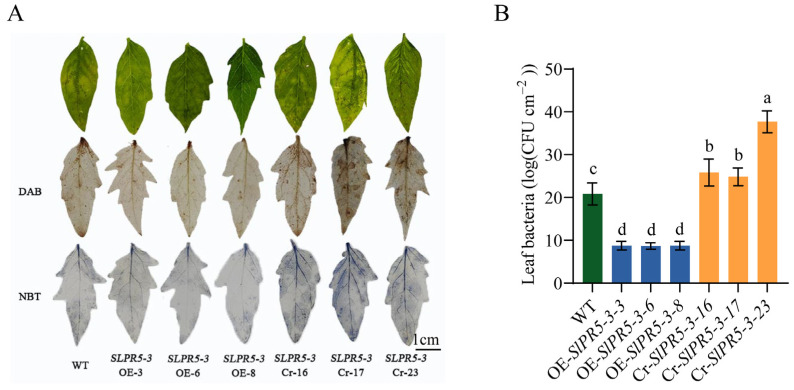
Changes in disease symptoms in *SlPR5-3*-overexpressing and CRISPR mutant tomato plants inoculated with *Pst* DC3000. (**A**) Leaf H_2_O_2_ and O_2_^▪−^ accumulation was observed by DAB and NBT staining 3 days after inoculation with *Pst* DC3000. (**B**) The number of bacteria on the leaves. Letters (a–d) indicate significant differences (*p* ≤ 0.05).

To investigate the function and role of *SlPR5-3*, which is strongly induced and expressed by *Pst* DC3000, in tomato resistance to bacterial leaf spot, *Pst* DC3000 inoculation assays were performed on one-month-old seedling-sized WT, OE-*SlPR5-3*, and Cr-*SlPR5-3* lines. Compared with the WT strain, the *SlPR5-3* CRISPR mutant strain showed a significant increase in the number of bacteria on the leaves (Figure 6B) and increased accumulation of H_2_O_2_ and O_2_^▪−^ compared with the WT (Figure 6A), whereas the overexpression strain showed a lower number of bacteria on the leaves, a decreased accumulation of H_2_O_2_ and O_2_^▪−^ and increased resistance to disease. These results suggest that *SlPR5-3* positively regulates disease resistance by either attenuating ROS damage or promoting ROS scavenging.

### 3.8. Measurement of Physiological Indicators of the Disease Resistance Response in SlPR5-3-Overexpressing and Mutant Lines

**Figure 7:**
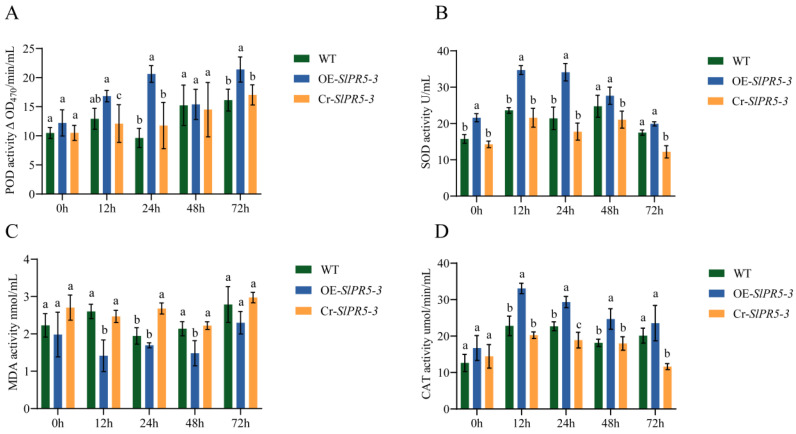
Changes in (**A**) POD, (**B**) SOD and (**D**) CAT activities and the (**C**) MDA content at different time points under *Pst* DC3000 stress conditions. Letters (a–c) indicate significant differences (*p* ≤ 0.05).

Physiological indices of three overexpression lines, three CRISPR-mutant lines and wild-type plants inoculated with the *Pst* DC3000 pathogen for 3 d were determined, as shown in Figure 4. The disease resistance of the *SlPR5-3* gene was determined by evaluating the SOD, POD, and CAT activities as well as the MDA content (Figure 7). The numerical data are shown in Table S3. Under the stress of bacterial leaf spot disease in tomato, the SOD, POD and CAT activities in the overexpression-treated plants were significantly higher than in the control, whereas they were decreased in the mutant lines (Figure 7). Compared with that in the WT strain, the MDA content in the overexpression strain was reduced and that in the CRISPR mutant strain was increased (Figure 7). The results revealed an increase in disease resistance in *SlPR5-3*-overexpressing plants and a decrease in disease resistance in CRISPR-mutant plants.

## 4. Discussion

In this study, all 27 *SlPR5* genes contained conserved structural domains for pathogen defense functions, and nine of the *SlPR5* genes expressed the thaumatin structural domain in tomato. The functions of these genes include three-dimensional structural stability, substrate binding and catalytic activity, and signal sensing and stress response [53], and 10 *SlPR5* genes contain a conserved TLP-PA structural domain. The functions of these genes are realized through four main types of mechanisms: structural stability, antipathogenic activity, the integration of stress signals and developmental regulation [54]. Structural domains associated with antifungal activity (TLP-PA) are present in some sequences of wild olive trees and have a potential role in pathogen resistance [55]. Seven *SlPR5* genes contain a conserved GH64-TLP-SF structural domain. The rice *OsTLP2* gene [56], the sugarcane *ScTLP2* and *ScTLP3* genes [57], and the *Arabidopsis AtTLP6* gene [58] all contain the GH64-TLP-SF structural domain, which plays a central role in pathogen defense and stress response [59,60]. These results suggest that *SlPR5* is involved in plant defense systems against biotic and abiotic stresses and in the regulation of physiological processes in many plant species.

By participating in transcriptional regulation, cis-acting elements play important roles in plant growth and development and the abiotic stress response [61], and the disease resistance functions of *SlPR5* family members are regulated by cis-acting elements in their promoter regions. These elements activate *SlPR5* gene expression in response to pathogen infestation, hormone signaling, and environmental stress through interactions with transcription factors. By analyzing the sequence structure of the internal and promoter regions of the genes, we found that all 27 *SlPR5* gene family members contain several different cis-acting elements and that W-box cis-acting elements are present in 11 *SlPR5* genes. The W-box responds directly to pathogenic effector molecules, activates the immune cascade response [62], and directly activates *SlPR5* expression. The W-box in the walnut *JrPR5L* promoter, which is bound and activated for expression by *JrWRKY21*, is involved in resistance to anthracnose [63]. The expression of W-box in the tobacco *NbTLP1* promoter is rapidly induced by *Mycobacterium avium* infection [64]. MYB is predicted to be present in 24 *SlPR5* genes, and both rice R2R3-MYB [65] and *Arabidopsis* AtMYB41 [66] contain MYB cis-elements and play regulatory roles in antiretroviral activity. Moreover, the MYB transcription factor plays a crucial role in the defense response of plants [67]. *SlPR5* also includes 13 response elements related to abiotic stresses, hormones and other inputs. The presence of promoter elements such as W-boxes and MYBs provides a structural basis for *SlPR5* family members to perform stress resistance regulatory functions, elucidating the possible modes of regulation of this gene family in the regulation of the stress response.

In this study, SlPR5 proteins were distributed in a variety of plant organs and were predominant in flower buds, the fruit epidermis, and roots, suggesting that the function of *SlPR5* genes may play an irreplaceable role in plant growth and development [68]. However, *SlPR5* genes may be localized differently and are predicted to be in extracellular regions [69] or to accumulate in vesicles in response to pathogen attack [70], depending on their specific function [71]. A similar report revealed that most of the PR5 expression in *Glycine max* is predicted to be in the extracellular region [72].

To explore the response pattern of the tomato *SlPR5* gene family to pathogen stress, we analyzed the expression of *SlPR5* gene family members under *Pst* DC3000 stress. The results revealed that most of the genes responded differently under *Pst* DC3000 stress conditions, while *SlPR5-3* responded most strongly. NbTLP1 is a homolog of PR5, and overexpression of NbTLP1 significantly enhanced chitin and flg22-induced ROS accumulation and strengthened the PTI response. Directly interacts with NbPR1 and stabilizes its protein level to enhance resistance to Phytophthora capsici through the SA pathway [64]. Potato StPR-5 may be involved in ROS burst and enhance resistance to late blight (Phytophthora infestans) by regulating NADPH oxidase (RBOH) activity [73]. Fls2 activates PTI, induces SA synthesis, and directly upregulates *SlPR5-3* expression [74,75]. Pto recognition of AvrPto triggers ETI and indirectly regulates *SlPR5-3* through the SA and ethylene pathways [76,77]. To further explore the role of *SlPR5-3* in resistance to *Pst* DC3000, reactive oxygen species and defense-related enzyme activities were measured in tomato plants infected with the pathogen. The accumulation of reactive oxygen species is considered one of the most direct antimicrobial effects and initiates signal activation of downstream defense responses [78]. Reactive oxygen species, including H_2_O_2_ and O_2_^▪-^, are the most important signaling molecules in plant immunity. The moderate accumulation of reactive oxygen species in plants is favorable for plant growth and development, but pathogenic bacteria increase the accumulation of reactive oxygen species in plants. Excessive accumulation of reactive oxygen species can exacerbate damage to plants and aggravate pathogen damage [79]. In soybean, *GmSTOP1-3* reduces ROS accumulation and increases aluminum tolerance [80]. The transcription of TaNOX10 by TaWRKY19 inhibited ROS production and enhanced wheat susceptibility to stripe rust [81]. In this study, H_2_O_2_ and O_2_^▪−^ accumulation were detected by DAB and NBT staining and quantification, respectively, in tomato inoculated with *Pst* DC3000 in both the wild-type and overexpressing materials, with the lowest amount of reactive oxygen species accumulation in the *SlPR5-3*-overexpression strain and significantly greater reactive oxygen species accumulation in the CRISPR-mutant *SlPR5-3* strain than in the wild-type strain.

In addition, we examined the activities of defense-related enzymes, the CAT, POD and SOD activities. The core function of defensive enzymes is the specific removal of ROS; the higher the enzyme activity, the more ROS are removed. The results showed that except for MDA, were significantly higher in *SlPR5-3*-overexpression plants than in the wild-type and CRISPR mutant lines after inoculation with *Pst* DC3000. After wheat was inoculated with *Pseudomonas syringae*, the damage caused by the bacteria to wheat leaves was delayed by the increased activity of defense enzymes, such as POD and SOD, and a decrease in the level of reactive oxygen species in the leaves [82]. The rice ubiquitin ligase RBRL enhances peroxidase (POD) and polyphenol oxidase (PPO) activity and inhibits viral replication [83]. High levels of defense-related enzyme activities and low levels of reactive oxygen species accumulation are detected in plant materials that are resistant to biotic stresses such as pathogens [84]. In summary, we hypothesize that *SlPR5-3* enhances tomato resistance to tomato bacterial leaf spot by decreasing ROS accumulation and increasing the activity of defense enzymes.

## 5. Conclusions

In this study, the tomato *SlPR5* gene family was analyzed at the genome-wide level, 27 *SlPR5* genes were obtained, and their phylogenetic relationships, physicochemical properties, gene structures, chromosomal locations, cis-acting elements, and conserved motifs were analyzed using bioinformatics methods. Disease resistance response was also analyzed on the basis of transcriptomic data, which revealed that most of the genes were able to respond to *Pst* DC3000 stress. The function of the *SlPR5-3* gene was verified by overexpression and knockdown treatments of the gene. Disease resistance analysis showed that *SlPR5-3* overexpression lines had reduced spots, lower pathogen counts, less ROS accumulation, and indicators such as SOD, POD, and CAT showed that plant disease resistance was improved compared with the wild type, whereas the gene-edited lines behaved in the opposite direction, with a significant decrease in disease resistance. *SlPR5-3* was found to have a positive regulatory effect under Pst DC3000 stress. These results deepen the research on the regulatory mechanism of the biotic stress response of the tomato *SlPR5* gene family and provide support for future innovative breakthroughs in tomato resistance breeding and the application of gene editing technology and are of great significance to the development of tomato disease resistance legacy breeding.

## Figures and Tables

**Table 1:** Physicochemical properties of the tomato SlPR5 family.

GENE ID	GENE NAME	Number of	Molecular	Theoretical	Aliphatic	GRAVY	Instability	Subcellular Localization
		Amino Acids (aa)	Weight (Da)	pI	Index		Index	
Solyc11g044400.1.1	SlPR5-1	87	9451.58	5.29	71.72	−0.301	41.54	Extracellular
Solyc08g080620.2.1	SlPR5-2	614	66,612.02	8.63	49.3	−0.411	36.49	Extracellular
Solyc08g080640.1.1	SlPR5-3	247	26,646.21	8.28	54.53	−0.246	35.52	Cytosol
Solyc08g080650.2.1	SlPR5-4	496	53,620.35	7.81	51.75	−0.293	39.47	Extracellular
Solyc07g017970.2.1	SlPR5-5	239	26,323.92	7.83	66.11	−0.147	31.17	Extracellular
Solyc01g086840.2.1	SlPR5-6	321	34,245.82	4.92	68.1	0.044	36.74	Chloroplast
Solyc01g111330.3.1	SlPR5-7	560	57,950.73	4.82	59.45	−0.065	37.49	Chloroplast
Solyc12g056390.2.1	SlPR5-8	878	97,494.25	5.9	57.97	−0.382	31.82	Vacuolar membrane
Solyc09g011027.1.1	SlPR5-9	253	27,399.66	8.46	72.02	−0.031	46.62	Chloroplast
Solyc02g087520.3.1	SlPR5-10	270	29,446.75	8.24	71.48	−0.093	54.51	Chloroplast
Solyc01g104290.2.1	SlPR5-11	318	34,394.2	6.83	75.69	−0.058	34.4	Cytosol
Solyc03g079960.3.1	SlPR5-12	297	31,399.74	4.82	71.92	0.112	44.84	Golgi apparatus
Solyc04g079890.3.1	SlPR5-13	247	25,388.45	4.99	63.72	0.156	29.89	Extracellular
Solyc10g084840.2.1	SlPR5-14	245	26,188.25	7.27	79.96	0.082	41.42	Extracellular
Solyc05g053020.3.1	SlPR5-15	244	26,027.97	8.02	79.96	0.084	34.5	Extracellular
Solyc04g007310.2.1	SlPR5-16	255	27,954.86	7.36	74.59	−0.121	27.31	Chloroplast
Solyc11g066130.1.1	SlPR5-17	252	27,265.05	8.15	70.87	−0.063	54.48	Extracellular
Solyc08g080585.1.1	SlPR5-18	237	25,973.4	8.13	51.1	−0.378	31.52	Extracellular
Solyc08g080670.2.1	SlPR5-19	472	51,865.27	6.07	46.29	−0.402	38.33	Chloroplast
Solyc06g073000.3.1	SlPR5-20	270	28,605.7	4.73	74.85	0.239	36.87	Chloroplast
Solyc08g080600.1.1	SlPR5-21	75	8266.26	5.09	59.87	−0.389	21.48	Chloroplast
Solyc02g083760.3.1	SlPR5-22	253	26,448.49	4.62	51.78	−0.13	44.18	Extracellular
Solyc04g081550.3.1	SlPR5-23	335	35,383.5	4.95	61.82	−0.145	42.72	Extracellular
Solyc02g083790.3.1	SlPR5-24	318	32,736.06	4.65	74.25	0.187	34.69	Chloroplast
Solyc11g013300.2.1	SlPR5-25	287	31,030.57	8.35	72.65	0.049	37.66	Extracellular
Solyc03g118780.3.1	SlPR5-26	245	26,121.88	8.92	63.02	−0.049	41.01	Chloroplast
Solyc03g033490.2.1	SlPR5-27	310	32,067.2	4.69	66.74	0.087	49.41	Extracellular

Note: Order is based on SGN number.

## Data Availability

Data are contained within the article and Supplementary Materials.

## Supplementary Materials

The following supporting information can be downloaded at: https://www.mdpi.com/article/10.3390/plants14213389/s1, Table S1: GFP-*SlPR5-3* gene PCR primers; Table S2: Primers for fluorescent quantitative qRT-PCR of tomato *SlPR5* gene family; Table S3: SOD, POD, CAT activities and MDA content; Table S4: Locus ID of the tomato *SlPR5* family; Figure S1: Comparative phylogeny of SlPR5-3 with other Solanaceae.

## References

[1] Yan Z.Y., Zhao M.S., Ma H.Y., Liu L.Z., Yang G. (2021). Biological and molecular characterization oftomato brown rugose fruit virus and development of quadruplex RT-PCR detection. J. Integr. Agric..

[2] Lindeberg M., Cartinhour S., Myers C.R., Schechter L.M., Schneider D.J., Collmer A. (2006). Closing the circle on the discovery of genes encoding Hrp regulon members and type III secretion system effectors in the genomes of three model *Pseudomonas syringae* strains. Mol. Plant-Microbe Interact..

[3] Yunis H., Bashan Y., Okon Y., Henis Y. (1980). Weather dependence, yield losses and control of bacterial speck of tomato caused by pseudomonas tomato. Plant Dis..

[4] Xu S., Zhang Z., Zhou J., Han X., Song K., Gu H., Sun L. (2022). Comprehensive analysis of NAC genes reveals differential expression patterns in response to Pst DC3000 and their overlap expression pattern during PTI and ETI in tomato. Genes.

[5] Hind S.R., Strickler S.R., Boyle P.C., Dunham D.M., Bao Z., O’Doherty I.M., Baccile J.A., Hoki J.S., Viox E.G., Clarke C.R. (2016). Tomato receptor Flagellin-Sensing 3 binds flgII-28 and activates the plant immune system. Nat. Plants.

[6] Roberts R., Liu A.E., Wan L., Geiger A.M., Hind S.R., Rosli H.G., Martin G.B. (2020). Molecular characterization of differences between the tomato immune receptors flagellin sensing 3 and flagellin sensing 2. Plant Physiol..

[7] Zhang N., Pombo M.A., Rosli H.G., Martin G.B. (2020). Tomato wall-associated kinase SlWak1 depends on Fls2/Fls3 to promote apoplastic immune responses to *Pseudomonas syringae*. Plant Physiol..

[8] Nguyen H.T., Daudi A., Shah J. (2010). Transcriptional reprogramming during establishment of systemic acquired resistance in Arabidopsis. Plant Physiol..

[9] Macho A.P., Zipfel C. (2014). Plant PRRs and the activation of innate immune signaling. Mol. Cell.

[10] Li L., Yu Y., Zhou Z., Zhou J.M. (2016). Plant pattern—Recognition receptors controlling innate immunity. Sci. China Life Sci..

[11] Dong J., Xiao F., Fan F., Gu L., Cang H., Martin G.B., Chai J. (2009). Crystal structure of the complex between *Pseudomonas* effector AvrPtoB and the tomato Pto kinase reveals both a shared and a unique interface compared with AvrPto-Pto. Plant Cell.

[12] Göhre V., Spallek T., Häweker H., Mersmann S., Mentzel T., Boller T., de Torres M., Robatzek S. (2008). Plant pattern-recognition receptor FLS2 is directed for degradation by the bacterial ubiquitin ligase AvrPtoB. Curr. Biol..

[13] Shan L., He P., Li J., Heese A., Peck S.C., Nürnberger T., Martin G., Sheen J. (2008). Bacterial effectors target the common signaling partner BAK1 to disrupt multiple MAMP receptor-signaling complexes and impede plant immunity. Cell Host Microbe.

[14] Kim Y.J., Lin N.C., Martin G.B. (2002). Two distinct Pseudomonas effector proteins interact with the Pto kinase and activate plant immunity. Cell.

[15] Nabi Z., Manzoor S., Nabi S.U., Wani T.A., Gulzar H., Farooq M., Vlădulescu C., Mansoor S. (2024). Pattern-Triggered Immunity and Effector-Triggered Immunity: Crosstalk and cooperation of PRR and NLR-mediated plant defense pathways during host–pathogen interactions. Physiol. Mol. Biol. Plants.

[16] Gu Y.Q., Wildermuth M.C., Chakravarthy S., Loh Y.T., Yang C., He X., Han Y., Martin G.B. (2002). Tomato transcription factors Pti4, Pti5, and Pti6 activate defense responses when expressed in Arabidopsis. Plant Cell.

[17] Sharma S., Bhattarai K. (2019). Progress in developing bacterial spot resistance in tomato. Agronomy.

[18] Fang X., Meng X., Zhang J., Cao S., Tang X., Fan T. (2021). AtWRKY1 negatively regulates the response of *Arabidopsis thaliana* to Pst. DC3000. Plant Physiol. Biochem..

[19] Zhang N., Hecht C., Sun X., Fei Z., Martin G.B. (2022). Loss of function of the bHLH transcription factor Nrd1 in tomato enhances resistance to *Pseudomonas syringae*. Plant Physiol..

[20] Li Y.M., Wang J., Wang P., Shi K. (2022). Function of sugar transport protein SlSTP2 in tomato defense against bacterial leaf spot. Sci. Agric. Sin..

[21] Wu Z., He L., Jin Y., Chen J., Shi H., Wang Y., Yang W. (2021). Histone Deacetylase 6 suppresses salicylic acid biosynthesis to repress autoimmunity. Plant Physiol..

[22] Van Loon L.C., Van Strien E.A. (1999). The families of pathogenesis-related proteins, their activities, and comparative analysis of PR-1 type proteins. Physiol. Mol. Plant Pathol..

[23] Piggott N., Ekramoddoullah A.K., Liu J.J., Yu X. (2004). Gene cloning of a thaumatin-like (PR-5) protein of western white pine (*Pinus monticola*, D. Don) and expression studies of members of the PR-5 group. Physiol. Mol. Plant Pathol..

[24] Sels J., Mathys J., De Coninck B.M., Cammue B.P., De Bolle M.F. (2008). Plant pathogenesis-related (PR) proteins: A focus on PR peptides. Plant Physiol. Biochem..

[25] Misra R.C., Sandeep, Kamthan M., Kumar S., Ghosh S. (2016). A thaumatin-like protein of *Ocimum basilicum* confers tolerance to fungal pathogen and abiotic stress in transgenic Arabidopsis. Sci. Rep..

[26] Eden D., Matthew J.B., Rosa J.J., Richards F.M. (1982). Increase in apparent compressibility of cytochrome c upon oxidation. Proc. Natl. Acad. Sci. USA.

[27] Dalen L.S., Johnsen Ø., Lönneborg A., Yaish M.W. (2015). Freezing tolerance in Norway spruce, the potential role of pathogenesis-related proteins. Acta Physiol. Plant..

[28] Liu C., Han L.H., Wang H.B. (2018). Identification and codon bias analysis of the sweet protein gene family in cereals. Northwest J. Agric..

[29] Anisimova O.K., Kochieva E.Z., Shchennikova A.V., Filyushin M.A. (2022). Thaumatin-like protein (TLP) genes in garlic (*Allium sativum* L.): Genome-wide identification, characterization, and expression in response to *Fusarium proliferatum* infection. Plants.

[30] Wei L., Zhang L., Wang W.W., Yu Z.Y., Yu D.Y., Liu L.J. (2016). Bioinformatics analysis of soybean disease course-related protein PR-5 and its homologous protein TPLs. Soybean Sci..

[31] Xi Z., Jia H., Li Y., Ma J., Lu M., Wang Z., Kong D.X., Deng W.W. (2024). Identification and functional analysis of PR genes in leaves from variegated tea plant (*Camellia sinensis*). Agronomy.

[32] Šimkovicová M., Kramer G., Rep M., Takken F.L. (2024). Tomato R-gene-mediated resistance against Fusarium wilt originates in roots and extends to shoots via xylem to limit pathogen colonization. Front. Plant Sci..

[33] Pressey R. (1997). Two isoforms of NP24: A thaumatin-like protein in tomato fruit. Phytochemistry.

[34] Anžlovar S., Dermastia M. (2003). The comparative analysis of osmotins and osmotin-like PR-5 proteins. Plant Biol..

[35] Li X., Xu B., Xu J., Li Z., Jiang C., Zhou Y., Zhao K. (2023). Tomato-thaumatin-like protein genes Solyc08g080660 and Solyc08g080670 confer resistance to five soil-borne diseases by enhancing β-1, 3-glucanase activity. Genes.

[36] Jia X., Zeng H., Wang W., Zhang F., Yin H. (2018). Chitosan oligosaccharide induces resistance to *Pseudomonas syringae* pv. tomato DC3000 in *Arabidopsis thaliana* by activating both salicylic acid–and jasmonic acid–mediated pathways. Mol. Plant-Microbe Interact..

[37] Weng Q.Y., Song J.H., Zhao Y.T., Zheng X., Huang C.C., Wang G.Y., Dong J.G. (2017). T1N6_22 positively regulates *Botrytis cinerea* resistance but negatively regulates *Pseudomonas syringae* pv. tomato DC3000 resistance in *Arabidopsis thaliana*. Biotechnol. Biotechnol. Equip..

[38] Khare E., Kim K., Lee K.J. (2016). Rice OsPBL1 (ORYZA SATIVA ARABIDOPSIS PBS1-LIKE 1) enhanced defense of *Arabidopsis* against *Pseudomonas syringae* DC3000. Eur. J. Plant Pathol..

[39] Jiang C.H., Fan Z.H., Xie P., Guo J.H. (2016). Bacillus cereus AR156 extracellular polysaccharides served as a novel micro-associated molecular pattern to induced systemic immunity to Pst DC3000 in Arabidopsis. Front. Microbiol..

[40] Fernandez-Pozo N., Menda N., Edwards J.D., Saha S., Tecle I.Y., Strickler S.R., Mueller L.A. (2015). The Sol Genomics Network (SGN)—From genotype to phenotype to breeding. Nucleic Acids Res..

[41] El-Gebali S., Mistry J., Bateman A., Eddy S.R., Luciani A., Potter S.C., Finn R.D. (2019). The Pfam protein families database in 2019. Nucleic Acids Res..

[42] Turkoglu M., Yanikoğlu B., Hanbay D. (2021). PlantDiseaseNet: Convolutional neural network ensemble for plant disease and pest detection. Signal Image Video Process..

[43] Swarbreck D., Wilks C., Lamesch P., Berardini T.Z., Garcia-Hernandez M., Foerster H., Huala E. (2007). The Arabidopsis Information Resource (TAIR): Gene structure and function annotation. Nucleic Acids Res..

[44] Gasteiger E., Gattiker A., Hoogland C., Ivanyi I., Appel R.D., Bairoch A. (2003). ExPASy: The proteomics server for in-depth protein knowledge and analysis. Nucleic Acids Res..

[45] Nordahl P.T., Søren B., Gunnar V.H., Henrik N. (2011). SignalP 4.0: Discriminating signal peptides from transmembrane regions. Nat. Methods.

[46] Horton P., Park K.J., Obayashi T., Fujita N., Harada H., AdamsCollier C.J., Nakai K. (2007). WoLF PSORT: Protein localization predictor. Nucleic Acids Res..

[47] Chen C., Chen H., Zhang Y., Thomas H.R., Frank M.H., He Y., Xia R. (2020). TBtools: An integrative toolkit developed for interactive analyses of big biological data. Mol. Plant.

[48] Bailey T.L., Boden M., Buske F.A., Frith M., Grant C.E., Clementi L., Ren J., Li W.W., Noble W.S. (2009). MEME SUITE: Tools for motif discovery and searching. Nucleic Acids Res..

[49] Zeng D.C., Ma X.L., Xie X.R., Zhu Q.L., Liu Y.G. (2018). Operational methods for plant CRISPR/Cas9 multi-gene editing vector construction and mutation analysis, Science in China. Life Sci..

[50] Bournonville C.F., Díaz-Ricci J.C. (2011). Quantitative determination of superoxide in plant leaves using a modified NBT staining method. Phytochem. Anal..

[51] Ramel F., Sulmon C., Bogard M., Couée I., Gouesbet G. (2009). Differential patterns of reactive oxygen species and antioxidative mechanisms during atrazine injury and sucrose-induced tolerance in *Arabidopsis thaliana* plantlets. BMC Plant Biol..

[52] Waese J., Fan J., Pasha A., Yu H., Fucile G., Shi R., Cumming M., Kelley L.A., Sternberg M.J., Krishnakumar V. (2017). ePlant: Visualizing and exploring multiple levels of data for hypothesis generation in plant biology. Plant Cell.

[53] Yang H.L., Yuan Z., Qian X. (2022). Expression Profile Analysis of Thionin-like Gene Family in Barley. Biotechnol. Bull..

[54] Wang T., Hu J., Ma X., Li C., Yang Q., Feng S., Li M., Li N., Song X. (2020). Identification, evolution and expression analyses of whole genome-wide TLP gene family in Brassica napus. BMC Genom..

[55] Alves M.C.S., de Souza R.S., da Silva R.C.C. (2024). Genome-Wide Identification and Characterization Thaumatin-like Protein (TLP) Genes in Wild Olive (*Olea europaea* var. sylvestris). Sci. Plena.

[56] Singh S., Tripathi R.K., Lemaux P.G., Buchanan B.B., Singh J. (2017). Redox-dependent interaction between thaumatin-like protein and β-glucan influences malting quality of barley. Proc. Natl. Acad. Sci. USA.

[57] Li C.N., Liu F., Zhang X., Ren Y.J., Tang H.C., Que Y.X. (2020). Bioinformatics and Expression Analysis of Thaumatin-like Protein Genes ScTLP2 and ScTLP3 from Sugarcane. Res. Gate.

[58] Zhang Y., He X., Su D., Feng Y., Zhao H., Deng H., Liu M. (2020). Comprehensive profiling of tubby-like protein expression uncovers ripening-related TLP genes in tomato (*Solanum lycopersicum*). Int. J. Mol. Sci..

[59] Sun L., Yu G., Han X., Xin S., Qiang X., Jiang L., Zhang S., Cheng X. (2015). TsMIP6 enhances the tolerance of transgenic rice to salt stress and interacts with target proteins. J. Plant Biol..

[60] Liu C., Han L., Wang H., Gao Y., Tang L. (2018). Research Advances on Plant Thaumatin-like Protein Family. Biotechnol. Bull..

[61] Baker S.S., Wilhelm K.S., Thomashow M.F. (1994). The 5′-region of Arabidopsis thaliana corl5a has cis-acting elements that confer cold-, drought- and ABA-regulated gene expression. Plant Mol. Biol..

[62] Gao Y., Zan X.-L., Wu X.-F., Yao L., Chen Y.-L., Jia S.-W., Zhao K.-J. (2014). Identification of fungus-responsive cis-acting element in the promoter of *Brassica juncea* chitinase gene, BjCHI1. Plant Sci..

[63] Zhou R., Dong Y., Liu X., Feng S., Wang C., Ma X., Liu J., Liang Q., Bao Y., Xu S. (2022). JrWRKY21 interacts with JrPTI5L to activate the expression of JrPR5L for resistance to *Colletotrichum gloeosporioides* in walnut. Plant J..

[64] Liu W., Zhang G., Liu C., Tian S., Qiao G., Sun H., Luo X., Wang S., Cai L., Sun X. (2025). NbTLP1 stabilizes NbPR1 to enhance resistance against *Phytophthora capsici* via salicylic acid signalling pathway in *Nicotiana benthamiana*. Plant Biotechnol. J..

[65] Zhang H.-C., Gong Y.-H., Tao T., Lu S., Zhou W.-Y., Xia H., Zhang X.-Y., Yang Q.-Q., Zhang M.-Q., Hong L.-M. (2024). Genome-wide identification of R2R3-MYB transcription factor subfamily genes involved in salt stress in rice (*Oryza sativa* L.). BMC Genom..

[66] Cominelli E., Sala T., Calvi D., Gusmaroli G., Tonelli C. (2008). Over-expression of the Arabidopsis AtMYB41 gene alters cell expansion and leaf surface permeability. Plant J..

[67] Liu Z., Luan Y., Li J., Yin Y. (2016). Expression of a tomato MYB gene in transgenic tobacco increases resistance to *Fusarium oxysporum* and Botrytis cinerea. Eur. J. Plant Pathol..

[68] Hu Z.L., Deng L., Yao N. (2009). Analysis of expression of the PR-1 and PR-5 genes in tomato. J. Southwest Agric. Univ..

[69] Samac D.A., Penuela S., Schnurr J.A., Hunt E.N., Foster-Hartnett D., Vandenbosch K.A., Gantt J.S. (2011). Expression of coordinately regulated defence response genes and analysis of their role in disease resistance in *Medicago truncatula*. Mol. Plant Pathol..

[70] Jami S.K., Anuradha T.S., Guruprasad L. (2007). Molecular, biochemical and structural characterization of osmotin-like protein from black nightshade (*Solanum nigrum*). J. Plant Physiol..

[71] Melchers L.S., Sela-Buurlage M.B., Vloemans S.A., Woloshuk C.P., Van Roekel J.S., Pen J., van den Elzen P.J., Cornelissen M.J. (1993). Extracellular targeting of the vacuolar tobacco proteins AP24, chitinase and β-1, 3-glucanase in transgenic plants. Plant Mol. Biol..

[72] Onishi M., Tachi H., Kojima T., Shiraiwa M., Takahara H. (2006). Molecular cloning and characterization of a novel salt-inducible gene encoding an acidic isoform of PR-5 protein in soybean (*Glycine max* [L.] Merr.). Plant Physiol. Biochem..

[73] Liu J., Hu Y., Lu X., Xu J., Wang H., Tang W., Li C. (2025). The role of ribosomal protein StRPS5 in mediating resistance of *Solanum tuberosum* plants to *Phytophthora infestans*. Plant Sci..

[74] Li J., Wen J., Lease K.A., Doke J.T., Tax F.E., Walker J.C. (2002). BAK1, an Arabidopsis LRR receptor-like protein kinase, interacts with BRI1 and modulates brassinosteroid signaling. Cell.

[75] Wang X., Zhang X., Liu Y., Ru L., Yan G., Xu Y., Yu Y., Zhu Z., He Y. (2025). miR398-SlCSD1 module participates in the SA-H_2_O_2_ amplifying feedback loop in *Solanum lycopersicum*. J. Adv. Res..

[76] Martin G.B., Brommonschenkel S.H., Chunwongse J., Frary A., Ganal M.W., Spivey R., Wu T., Earle E.D., Tanksley S.D. (1993). Map-based cloning of a protein kinase gene conferring disease resistance in tomato. Science.

[77] Van Loon L.C., Rep M., Pieterse C.M.J. (2006). Significance of inducible defense-related proteins in infected plants. Annu. Rev. Phytopathol..

[78] Craig A., Ewan R., Mesmar J., Gudipati V., Sadanandom A. (2009). E3 ubiquitin ligases and plant innate immunity. J. Exp. Bot..

[79] Govrin E.M., Levine A. (2000). The hypersensitive response facilitates plant infection by the necrotrophic pathogen *Botrytis cinerea*. Curr. Biol..

[80] Liu G., Li D., Mai H., Lin X., Lu X., Chen K., Wang R., Riaz M., Tian J., Liang C. (2024). GmSTOP1-3 regulates flavonoid synthesis to reduce ROS accumulation and enhance aluminum tolerance in soybean. J. Hazard. Mater..

[81] Wang N., Fan X., He M., Hu Z., Tang C., Zhang S., Lin D., Gan P., Wang J., Huang X. (2022). Transcriptional repression of TaNOX10 by TaWRKY19 compromises ROS generation and enhances wheat susceptibility to stripe rust. Plant Cell.

[82] Zhang M., Kang H., Zhang G., Chen Y., Kong X., Guo Q., Wang W.J.P. (2015). Overexpression of TaUb2 enhances disease resistance to *Pseudomonas syringae* pv. tomato DC3000 in tobacco. Physiol. Mol. Plant Pathol..

[83] Huang Y., Yang J., Sun X., Li J., Cao X., Yao S., Han Y., Chen C., Du L., Li S. (2025). Perception of viral infections and initiation of antiviral defence in rice. Nature.

[84] De Gara L., de Pinto M.C., Tommasi F. (2003). The antioxidant systems vis-à-vis reactive oxygen species during plant–pathogen interaction. Plant Physiol. Biochem..

