# Supplementary material for: Bioinformatics Analysis of the Tomato SlPR5 Gene Family and the Thaumatin-like Protein SlPR5-3 Positively Regulates Tomato Resistance to Pst DC3000
Source: Plants (Basel). 2025 Nov 5;14(21):3389. doi: 10.3390/plants14213389 (PMC12608184; doi:10.3390/plants14213389)
Supplement: Supplementary file 1 [file plants-14-03389-s001.zip › plants-3947142-supplementary.pdf]

Table S1. GFP-*SlPR5-3* gene PCR primers.

Primer name	Forward primer 5′→3′	Reverse primer 5′→3′
GFP-SlPR5-3	CGGGGGACGAGCTCGGTACCATG GGCTACTTGACATCTTC	GGTGTCGACTCTAGAGGATCCA CTTGGCCACTTCATCAG
CRISPR target validation	CACCCCTATATAAAGTGCTTTC	CATTTATATTGGCCGTGCAATG

Table S2. Primers for fluorescent quantitative qRT-PCR of tomato *SlPR5* gene family

Primer name	Forward primer 5′→3′	Reverse primer 5′→3′
SlPR5-3	ATACTTATGCTGCCACTATTGAGGT	TGTAAGACTCCACCACAATCACC
SlPR5-4	TACCACGTTCGGAGGACAAC	AGCCTCTTCATCACTTGAGGG
SlPR5-19	TTTCGGCCCAACTAACCCTG	ACCACATGGACCTTGAGTGC
SlPR5-17	TTCACTCACTTACCCACCGC	GCGGAGACGAACATTCATGC
SlPR5-23	GTGTGGTGGATTTGAACGGC	AGCTGACGATGCAACTGGAA
SlPR5-10	GAGACTGGAGTGGACGCATT	GAGTGTAGCTGGAGGAAGGC
Actin-7	ACTGGAATGGTGAAGGCTGG	CCCAGTTGCTGACAATACCG

Table S3. SOD, POD, CAT activities and MDA content

	WT	OE-*SLPR5*	Cr-*SLPR5*
CAT-0h	12.64±1.35	16.73±1.97	14.43±1.86
CAT-12h	22.80±1.54	33.07±0.82	20.26±0.52
CAT-24h	18.15±0.58	24.68±1.62	17.97±1.07
CAT-48h	22.68±0.70	29.35±0.88	18.88±1.24
CAT-72h	20.12±1.18	23.56±2.80	11.62±0.47
MDA-0h	2.22±0.18	1.98±0.34	2.71±0.19
MDA-12h	2.60±0.11	1.41±0.24	2.46±0.09
MDA-24h	2.13±0.11	1.48±0.19	2.22±0.06
MDA-48h	1.94±0.12	1.69±0.03	2.67±0.08
MDA-96h	2.78±0.27	2.29±0.17	2.97±0.08
SOD-0h	15.74±0.69	21.59±0.64	14.25±0.52
SOD-12h	23.60±0.45	34.72±0.70	21.59±1.50
SOD-24h	24.75±1.74	27.65±1.36	21.06±1.35
SOD-48h	21.42±1.79	34.11±1.38	17.75±1.35
SOD-96h	17.49±0.42	19.91±0.33	12.20±0.96
POD-0h	10.48±0.54	12.22±1.29	10.50±0.75
POD-12h	12.93±1.03	16.81±0.56	12.10±1.86
POD-24h	15.24±2.00	15.40±1.49	14.50±2.70
POD-48h	9.63±0.95	20.63±0.83	11.76±2.29
POD-96h	16.13±1.08	21.40±1.24	17.03±0.99

Table S4. Locus ID of the tomato *SlPR5* family.

GENE ID	GENE NAME	LOCUS ID
Solyc11g044400.1.1	*SLPR5-1*	XM_049519602
Solyc08g080620.2.1	*SLPR5-2*	NM_001247232
Solyc08g080640.1.1	*SLPR5-3*	NM_001305993
Solyc08g080650.2.1	*SLPR5-4*	NM_001309287
Solyc07g017970.2.1	*SLPR5-5*	XM_027918171
Solyc01g086840.2.1	*SLPR5-6*	XM_004229555
Solyc01g111330.3.1	*SLPR5-7*	XM_004230976
Solyc12g056390.2.1	*SLPR5-8*	XM_010316183
Solyc09g011027.1.1	*SLPR5-9*	XM_016699842
Solyc02g087520.3.1	*SLPR5-10*	XM_004233643
Solyc01g104290.2.1	*SLPR5-11*	XM_010316538
Solyc03g079960.3.1	*SLPR5-12*	XM_004234997
Solyc04g079890.3.1	*SLPR5-13*	XM_004238172
Solyc10g084840.2.1	*SLPR5-14*	XM_004249492
Solyc05g053020.3.1	*SLPR5-15*	XM_006355637
Solyc04g007310.2.1	*SLPR5-16*	XM_015216877
Solyc11g066130.1.1	*SLPR5-17*	NM_001247785
Solyc08g080585.1.1	*SLPR5-18*	XM_060351127
Solyc08g080670.2.1	*SLPR5-19*	NM_001247422
Solyc06g073000.3.1	*SLPR5-20*	XM_004241384
Solyc08g080600.1.1	*SLPR5-21*	XM_069287820
Solyc02g083760.3.1	*SLPR5-22*	XM_004232450
Solyc04g081550.3.1	*SLPR5-23*	XM_004238318
Solyc02g083790.3.1	*SLPR5-24*	XM_004232449
Solyc11g013300.2.1	*SLPR5-25*	XM_004250295
Solyc03g118780.3.1	*SLPR5-26*	XM_004235762
Solyc03g033490.2.1	*SLPR5-27*	XM_069296277

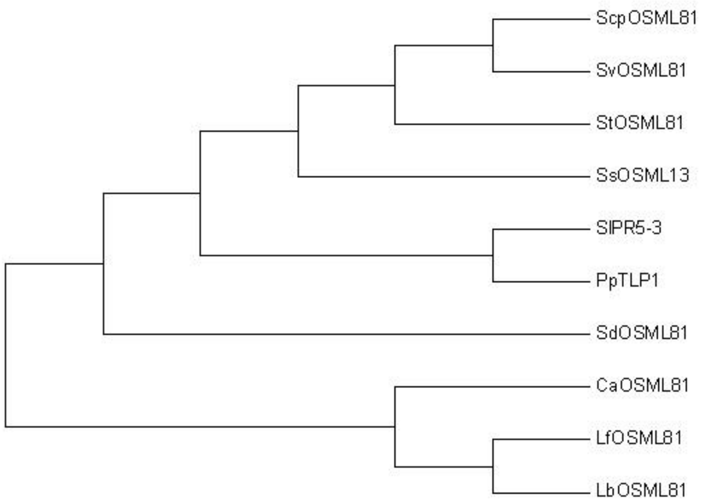

Figure S1. Comparative phylogeny of SlPR5-3 with other Solanaceae
